# Evasive entrepreneurship: Circumventing and exploiting institutional impediments for new profit opportunity in an emerging market

**DOI:** 10.1371/journal.pone.0247012

**Published:** 2021-02-19

**Authors:** Nnaoke Ufere, James Gaskin

**Affiliations:** 1 iServiceX, Inc. & Case Western Reserve University, Marietta, Georgia, United States of America; 2 Brigham Young University, Provo, Utah, United States of America; National Institute of Public Finance and Policy, INDIA

## Abstract

Evasive entrepreneurship (circumvention and exploitation of institutions by entrepreneurs) is a prevalent practice in many developing economies. Extant literature on the topic falls short of providing adequate theories to explain its triggers, mechanisms, and consequences. Leveraging extensive survey data from the World Bank, we used structural equation modeling to examine the relationship between evasive entrepreneurial behavior—tax evasion and bribery—and the relative payoff of such practices. Of the 2599 Nigerian entrepreneurs in our sample, the majority admitted to engaging in evasive entrepreneurship. The data suggest that institutional factors thought to constrain entrepreneurship in emerging markets are counter-intuitively perceived by founders as opportunities to earn large rents and improve firm performance. Our results emphasize the urgent need to eliminate institutional constraints that paradoxically enable the growth of evasive entrepreneurship in emerging economies. Our results also suggest that prevailing local conventions involving evasive behavior may motivate nascent entrepreneurs to imitate bribery and tax evasion, normalizing malfeasance as ‘best practice.’

## Introduction

Despite a large and engaging literature extolling productive entrepreneurship and its positive effects on economic growth, innovation and job creation, there is a deficit of research about evasive entrepreneurship and its consequences. Boettke and Coyne [1], Coyne and Leeson [2] and Elert and Henrekson [3] introduced the concept of evasive entrepreneurship to capture entrepreneurial practices aimed at circumventing existing institutional framework in different economies. Elert and Henrekson [3] characterize acts of evasive entrepreneurship as productive, unproductive, or destructive–advancing prior seminal work by Baumol [4]. In the Schumpeterian perspective, an evasive entrepreneur is a rule-breaker who purposively evades rules, regulation and norms to gain an advantage relative to other actors in the market [5]. The evasive entrepreneur thrives when institutional impediments to entrepreneurial activities are cumbersome [6, 7].

Many studies [4, 8–13] have shown that evasive entrepreneurship is common where taxation is high and regulations are cumbersome or where rule of law is unstable and enforcement is weak and doesn’t provide the necessary certainty for exchange and investment to take place (e.g., where property rights, thus understood, are weak or uncertain). What is unclear from extant literature is the institutional conditions that are likely to trigger circumvention, mechanisms through which entrepreneurs circumvent and whether enactment of evasive behavior is likely to lead to new profit opportunities. Why would entrepreneurs merely evade institutional impediments if they can exploit them for profits? What role do formal (e.g., legal, regulative, economic) and informal (e.g., customs, cultural norms) institutions play in incentivizing evasive entrepreneurial behavior? Does the effect of evasive behavior on venture performance differ between opportunity and necessity entrepreneurship? Opportunity-driven entrepreneurs start new firms based on the discovery of unexploited or underexploited business opportunities while necessity-driven entrepreneurs do so because they lack other income options [14].

Evasive entrepreneurship research is therefore limited by these and other fundamental, unanswered questions, for which there does not exist a cohesive explanatory or predictive theory. This state of research is surprising given the documented extent of evasive entrepreneurship–especially in emerging economies, such as Africa–and spirited calls for academic inquiry about them [3, 9, 15, 16]. As one example of this prevalence, in a qualitative study based on semi-structured interviews, Nigerian entrepreneurs self-identified as active collaborators rather than victims in evasive bribery schemes with significant upside potential for their firms [7].

There is a paucity of market-specific empirical research on the nexus of evasive entrepreneurship and institutional context [2, 3, 9]–and, to our knowledge, no empirical link between institutional constraints, evasive and exploitative behavior by entrepreneurs and firm performance, although there is some evidence that higher performance for entrepreneurs is linked to being more ethical and productive in general [17, 18]. This gap in the literature has further motivated our investigation of evasive entrepreneurship–specifically manifested as bribery and tax evasion − by founders in an emerging economy. We examine evasive entrepreneurship with respect to formal entrepreneurs (registered in official government statistics) embedded in both formal (legal, regulative and economic) and informal (cultural norms) institutions in Nigeria, the evasion mechanism and its consequences on firm performance. Here we view entrepreneurship as an embedded socio-economic process [6, 19]. In addition to the formal rules of the games that regulate economic activities, entrepreneurs draw from the social context in which they are embedded which shapes entrepreneurial behavior and outcomes.

To address this gap, we explore the general research question: What is the performance implication of evasive entrepreneurship in a developing country? This general question can be further broken up into these important and more granular questions identified above as missing from extent literature. We pursue these questions by examining the 2009 World Bank Enterprise Survey (WBES) data, collected from 2599 firms, to shed light on the nature and extent of evasive practices by entrepreneurs, and factors that contribute to its prevalence and persistence.

## Prevalence of evasive entrepreneurship among Nigerian SMEs

Nigeria is one of the world’s highest ranked nations in terms of corruption and tax evasion [20, 21] and has a large number of SMEs [22] that operate in many sectors (manufacturing, retail, services, etc.,) and account for well over 50% of Nigerian GDP [23] as well as an estimated 70% of industrial employment [24]. According to the 2009 WBES, over 50% of registered Nigerian entrepreneurs can be classified as evasive entrepreneurs because they pay bribes and evade taxes to circumvent or exploit institutional constraints. The majority of firms in our sample admitted engagement in both bribery and tax evasion. Bribery is often evidenced as illegal payment to officials to circumvent institutionalized due processes and to secure government services (e.g., utilities, permits and licenses) and/or lucrative contracts. Tax evasion manifests as under-reporting revenue and labor to evade income and wage taxes mandated by the government.

Entrepreneurs who own SMEs enjoy greater opportunity for bribery and tax evasion than larger firms and seem largely undeterred by the risk of detection and payment of penalties [25]. The widespread existence of evasive entrepreneurship does not mean that there is a complete absence of productive, innovative entrepreneurial activity in Nigeria [7].

Failing to pay taxes by under-reporting revenues or wages associated with tax payments is illegal in Nigeria. Intentional tax evasion is pervasive in Nigeria because much of the nation’s economic activity takes place in SMEs or is conducted by individual entrepreneurs in retail and service businesses [26, 27]; the activities of which are difficult to track in the larger economy. Tax evasion in Nigeria has been attributed to perceived high taxation, irregularity of tax administration and a corrupt tax administration culture in which tax assessors and collectors enable evasion by collecting bribes instead of taxes owed [25, 27] and to a self-interested judiciary and loose law enforcement system which lower the risk of adverse consequences [26]. In Nigeria, there are over 500 taxes and levies imposed on entrepreneurs by various tiers of government. For this reason, the 2019 World Bank Doing Business Report [28], ranked Nigeria 157^th^ out of 190 countries with regard to the ease of paying taxes. Nigeria was also ranked 184^th^ for registering new property, 182^nd^ for trading across borders, and 171^st^ for getting electricity. At the national level, tax evasion reduces revenue to fund critically needed social and economic development programs and, at the firm level, competition is impeded if tax evaders can undersell taxpayers [29].

Bribery is endemic in Nigeria, evidenced by its ranking on scales published annually by Transparency International (TI), an international NGO dedicated to raising public awareness of corruption. On its 2018 Corruption Perception Index, TI ranked Nigeria 144^th^ of 180 nations. While general causes of bribery in developing markets have been well-researched [e.g., 57], scant attention has been paid to how entrepreneurs use bribery to evade or exploit institutional impediments. Scholars, however, have suggested (though we cannot find any who have empirically tested) that in highly-regulated and bureaucratic environments like Nigeria, corruption may counterintuitively have positive financial impacts on the firms of evasive entrepreneurs by allowing them to bypass bureaucracy [30].

Intense competition for lucrative government contracts, permits, and licenses has long fueled bribery in Nigeria [31, 32] where participation in government-issued requests for proposals for procurement contracts is a lengthy and cumbersome process. Firms reportedly dedicate resources and time to mingle with government officials or their representatives, during which corrupt behavior often manifests [31, 33].

## Theoretical background

Entrepreneurship has been characterized from the perspective of innovation [34], arbitrage [35], and the nexus of opportunity and agency [36]. However, for the purpose of our study, we are most interested in understanding entrepreneurship as the exercise of agency to discover, evaluate, and exploit profit opportunities [35, 36]. By adopting this view, we define evasive entrepreneurship as the exercise of agency to circumvent or exploit institutional impediments to make a profit. Examples of evasive behavior include tax evasion and using bribe payment to circumvent cumbersome regulation and bureaucratic demands on business operations [2, 7]. A cross-national study by Ayyagari, Demirguc-Kunt [37] based on 2005 World Bank data, revealed that up to 30% of small and medium sized enterprises (SMEs) in Africa, Asia, Europe, Latin America and the Middle East pay bribes and evade taxes.

Implicit in the emphasis scholars and policy makers ascribe to entrepreneurship in emerging and transition economies [14] is the notion that it will be conducted legally and morally [8]. Furthermore, it is assumed that this well-behaved entrepreneurialism will naturally translate into national economic growth [14]. In this context, policy emphasizes facilitating the development and growth of small and medium-sized firms and entrepreneurship. Such policy has become increasingly important in national and regional development planning in developing economies. For example, Nigeria and several Sub-Saharan African countries have adopted national economic policies aimed to transition from a natural, resource-based economy to an entrepreneurial and taxable economy [38, 39] to generate economic growth and social progress.

Under certain conditions, however, entrepreneurial practices may reduce rather than enhance economic development. Baumol [4], for example, famously theorized that founders may enact productive or unproductive–and even destructive—roles based on the strength or weakness of a firm’s institutional context and “the rules of the game” that govern the rewards of one entrepreneurial activity relative to another. Evasive entrepreneurship embodies both unproductive and destructive roles [2, 3]. Some scholars have suggested that evasive acts may be productive. The justification of the productive effects rests on the so-called “grease the wheels” hypothesis [30, 40–42]. The hypothesis suggests that evasive behavior may be efficiency enhancing in a second-best context because it enables entrepreneurs to overcome inefficiencies caused by institutions.

Institutional theorists [e.g., 43–45] argue that entrepreneurship is institutionally constrained because institutions (formal and informal) create and shape opportunities (legal and illegal) for business owners and incentivize their exploitation. Baumol’s seminal work [4] contributed to the literature by theorizing that institutions determine not only the opportunity set, but also the type of entrepreneurship. Individuals allocate their entrepreneurial talent to productive, unproductive or destructive activities depending on the relative returns. However, Elert and Henrekson [3] argue that institutions do not merely shape entrepreneurial behavior, entrepreneurs may shape institutions through rent-seeking evasive practices. We also draw from social cognitive theory of emergent interactive agency [46] to examine how evading entrepreneurs exercise agency to influence and exploit institutions’ impediments for profit-making.

### Research model

**Fig 1:**
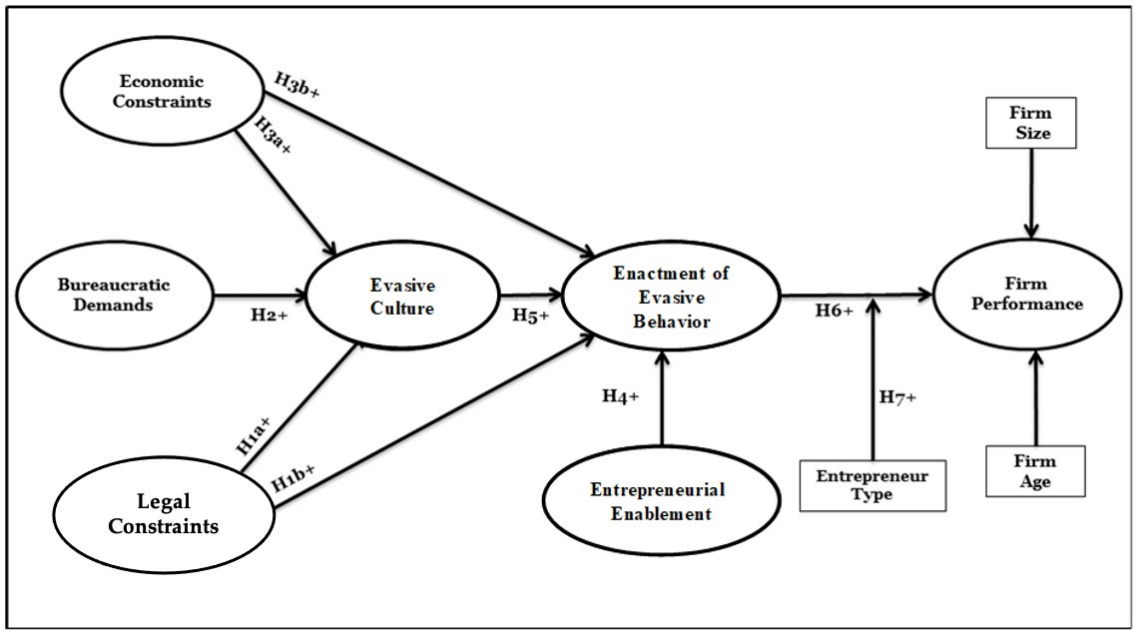
Conceptual model.

We conjectured that financial performance of small and medium sized firms in Nigeria will vary by the extent to which they engage in evasive entrepreneurship—enacting bribery and tax evasion to evade institutions—such that the more the firm engages in evasive acts, the higher performance it will achieve. As summarized in Fig 1, we propose evasive entrepreneurship is influenced by four factors: evasive culture, level of agency enabled by the entrepreneur, and extent of legal and economic constraints to doing business. We conjecture that evasive culture will be driven by three factors: bureaucratic demands exerted on entrepreneurs, and economic and legal constraints placed on entrepreneurial activities.

## Hypothesis development

### Legal constraints and evasive behavior

We define legal constraints as the degree to which the legal system and law enforcement are obstacles to doing business. Legal constraints can create a culture conducive for individuals to act evasively by influencing beliefs about the benefits and risks of unlawful behavior [47]. In Nigeria, the legal system is underdeveloped and weak, courts are inefficient and costly to use, which causes firms to engage in extra-legal activities such as bribery and tax evasion to overcome legal institutional constraints and business uncertainties [7, 48]. Moreover, firms are more likely to behave evasively if the legal system is ineffective and perceived as corrupt and unfair [49]. When a legal system works, it imposes risks both on those who demand bribes and those who pay them [50], while a dysfunctional system facilitates evasion and illegality with impunity [25]. A legal institution characterized by low probability of detection and concern about property right protection, contract enforcement and dispute resolution may also promote or enable unlawful and evasive behavior [49]. Thus, we hypothesize:

Hypothesis 1a. Legal constraints increase the perception of evasive culture.

Hypothesis 1b. Legal constraints increase the enactment of evasive behavior.

Regarding the effect running from legal constraints to culture, there is likely a bidirectional relationship (laws reflect culture à la Hayek), in that culture can also affect the law. According to Williamson [51], cultural change even occurs on a much longer time-scale than formal institutions, and while one can certainly argue with that (see e.g. McCloskey and Behymer [52]), it is undoubtedly the case that discrepancies between law and culture (institutional incongruence) can help further evasive behavior (see e.g. Elert and Henrekson [3]). Nevertheless, we chose the direction that we hope will make the most sense to most readers.

### Bureaucratic demand and evasive culture

We define bureaucratic demand as the level of regulatory demands placed on entrepreneurial activity. In a cross-national study of 30 developed and developing countries, Picur and Riahi‐Belkaoui [53] found tax evasion and corruption highest in countries with large regulatory bureaucracies. Similarly, countries with heavier regulation of entry have higher corruption and larger unofficial economies [54]. Regulation is a particularly important policy instrument employed by governments in developing markets to control firm behavior and combat market failures—but is pervasively exploited in countries with weak governance where officials with regulatory responsibility are provided wide discretionary power [55] and wield it to control market entry (by, for example, managing permits and licenses) and engage in excessive intervention in firm operations [56].

In Nigeria, 84% of entrepreneurs report spending up to 30% of their time dealing with government requirements—many imposed by rent-seeking bureaucrats at local, state and/or federal levels [56, 57]. Oppressive rules and regulations, with high compliance costs, impose disproportionate burdens on small and medium-sized firms, incentivizing entrepreneurs to act evasively (e.g., offer bribes to secure contracts or to avoid regulatory demands) to overcome red tape and ensure firm survival—rather than spending a lot of unproductive time on compliance [58]. Thus, we hypothesize:

Hypothesis 2. Bureaucratic demands increase the perception of an evasive culture.

### Economic constraints and evasive culture

We define economic constraints as the extent of financial and tax obstacles faced by entrepreneurs and their firms. WBES [57] data suggest Nigerian entrepreneurs perceive the following as obstacles to doing business: access to financing (75%), costs of financing (76%), tax rates (72%) and tax administration requirements (61%). Only 4% of Nigerian entrepreneurial firms have lines of credit or loans from financial institutions. Thus, Nigerian entrepreneurs face severe economic constraints. Several empirical studies [59, 60] demonstrate firms with limited access to resources (i.e., economic constraints) are more likely to engage in evasive behavior to ensure survival. Okpara and Wynn [61], for example, suggest that financial constraints may motivate evasive or illegal acts to secure bank financing or subsidized loans. Besley and McLaren [62] showed entrepreneurs will bribe tax collectors to alleviate tax burden and conserve cash flow. Informed by empirical evidence linking economic constraints with a propensity to act evasively, we hypothesize:

Hypothesis 3a. Economic constraints increase the perception of an evasive culture.

Hypothesis 3b. Economic constraints increase the enactment of evasive behavior.

### Enablement of entrepreneurial agency and evasive behavior

We define entrepreneurial agency as the level of intentional and purposive action taken by an entrepreneur to discover, evaluate, decide and exploit profit opportunities created by the institutional environment. Bandura [46] argues that individuals exercise agency based on beliefs about self-efficacy, driven by goal achievement and motivated by the expected rewards from action. Enablement of entrepreneurial agency, therefore, is a multidimensional construct in both concept and measurement. The entrepreneur’s personal self-efficacy advantage to capture profit opportunity lies in ability to process information, and particularly to synthesize information to enable identification and evaluation of profit opportunities. This requires considerable judgement and knowledge of the institutional context and the relative payoff of evasive acts. The link between agency and individual characteristics has been well studied. Propensity to enable personal agency has been linked to: pressure to perform [63, 64]; goal orientation [46]; age [65], tenure and experience [66], educational attainment [67], position in the firm [68], and share of firm equity ownership [16]. We argue that enablement of entrepreneurial agency is important in understanding evasive behavior in institutionally constrained context.

Further, the literature on choice behavior [69] suggests that an entrepreneur’s decision to engage in evasive behavior will involve deliberate evaluation of the corruptness of the culture, including assessment of associated risks, costs and benefits that will ‘arouse’ the entrepreneur to action [70] quite rationally. Therefore, in an environment that fosters, or fails to deter, illegal behavior, entrepreneurs will engage in evasive behaviors when the expected benefits exceed perceived costs. Consequently, we suggest that enablement of entrepreneurial agency to evade institutional constraints will reflect their knowledge of the culture, propensity for risk, exposure to information that allows calculation of the costs (both monetary and social), benefits to be derived, and competence to enact. Thereby, an entrepreneur becomes more empowered to act evasively. We hypothesize:

Hypothesis 4. The extent of enablement of entrepreneurial agency will increase the enactment of evasive behaviors.

### Evasive culture and evasive behavior

Scholars have long suggested that culture influences individual decision-making to enact illegal and evasive behavior [71–75]. This is a fairly logical relationship, and essentially a replication, but we include it to complete our model. The choice and propensity to act illegally depends on the cultural acceptance and supply of attractive evasive opportunities as well as their distribution [76], the trade-off of cost vs. gains [77], and individual moral character [67]. We hypothesize:

Hypothesis 5. A more evasive culture will increase the enactment of evasive behaviors.

### Evasive behavior and firm performance

How entrepreneurial evasive practices affect firm performance is a relatively unexplored area of inquiry. Rose-Ackerman [49, 78, 79] has suggested that it depends on benefits received in return. Given Nigeria’s corrupt legal system and weak law enforcement [61, 80], the probability of detection, prosecution and punishment are low. Therefore, from the perspective of the evasive entrepreneur, the costs of illegal evasive practices will be relatively low compared to its benefits. Because excess returns flow to the entrepreneur’s firm, perhaps somewhat counterintuitively we contend that the enactment of evasive behavior by entrepreneurs in Nigeria will positively impact overall firm performance.

In a previous qualitative study based on semi-structured interviews, Nigerian entrepreneurs self-identified as active collaborators rather than victims in evasive bribery schemes with significant upside potential for their firms [7]. Similarly, Hellman, Jones [81] studying the effect of state capture by firms, reported those that paid bribes to win government contracts increased firm performance relative to firms that did not. In a systematic review of management literature on the effect of firm-level corruption on firm performance, Galang [82] posits that differences in adaptation capacity and purposive strategic response to institutional corruption allow some firms to benefit from it more than others. In Nigeria, firms regularly engage in evasive transactions to secure licenses that assure monopoly or competitive advantage [80], as well as to win contracts with significant upside potential [31, 33], and collude with tax collectors to reduce tax payments. Accordingly, we hypothesize (specifically in the context of low-enforcement countries):

Hypothesis 6. The enactment of evasive behavior will increase firm performance.

### Moderating effect of entrepreneur type

Scholars differentiate ‘opportunity’ and ‘necessity’ entrepreneurship [14]. Opportunity-driven entrepreneurs start new firms based on the discovery of unexploited or underexploited business opportunities while necessity-driven entrepreneurs do so because they lack other income options [14]. Opportunity entrepreneurs are more growth-oriented, risk tolerant and educated than necessity entrepreneurs who are in it for subsistence. We expect the differences between opportunity and necessity entrepreneurship will affect propensity for evasive behavior and its effect on performance. Therefore, we hypothesize:

Hypothesis 7. The strength of the positive relationship between enactment of evasive behavior and firm performance will be stronger for necessity entrepreneurs versus opportunity entrepreneur.

## Methodology

### Data source

The WBES [57], our source of data, involves a stratified sample of manufacturing, retail and service firms globally. Using standardized survey instruments and a uniform sampling procedure to minimize measurement error and yield data comparable across economies, these surveys offer the most accurate global data available. The WBES for Nigeria has several unique advantages that make it suitable for investigating the relationship between evasive entrepreneurial behavior and firm performance. It provides not only data on entrepreneurs’ equity ownership status, role in the firm, experience, education, tenure and age, but detailed information on the bureaucratic demands and institutional and economic constraints they face, as well as self-reports of their bribe payment and tax evasion practices.

Sensitive questions about evasive practices are scattered in the survey to minimize potential respondent reticence. While a major concern with self-reported evasive behaviors such as bribery and tax evasion is whether reliable data can be collected given the secretive nature of the transactions, WBES informants candidly reported it. To avoid implicating respondents, emotionally charged words such as ‘corruption,’ ‘bribe’ and ‘tax evasion’ did not appear in the survey. Rather, respondents were asked, for example, ‘when establishments like this one do business with the government, what percentage of the contract value would typically be paid in informal payments to secure the contract?’ The Nigerian survey was conducted in cooperation with respected business organizations and industry associations, legitimizing it to respondents, thus minimizing the potential for non-response and false response [83].

### Description of the Nigerian sample

The Nigerian sample consisted of small and medium sized enterprises identified as such in the survey questionnaire. Small private firms were those characterized by less than 20 workers, medium private firms were those with 20 to 100 workers, and large private firms were those with more than 100 workers. Although the WBES sample consisted of a total of 3157 cases, we extracted 1,939 small and 660 medium sized firms for further analysis since they met our criteria as SME government registered entities operating in the formal economy. We focus our analysis on formal entrepreneurship—those officially registered in Nigerian government statistics. Whereas informal entrepreneurs operate in the shadow economy outside the purview of the government. A recent study by Ogbuabor and Malaolu [84] estimates that the size of the informal economy is about 64.6% of GDP. We used firm size and age as control variables to account for their effects across firms and groups.

As shown in Table 1, 49% (1274) were manufacturing, 22% (565) retail and 29% (760) service industry firms. Eighty-two percent of respondents (2125) founded and managed their firms and 92% (2508) owned majority share (>51%) in the firm. The other 18% were top executives but not founders. Females (354) constituted 14% of the sample.

**Table 1:** Summary description of sample (n = 2599).

Entrepreneur	Size	%	Firm and Environment	Size	%
**Gender**			**Firm Size**		
Female	354	14%	Small	1939	75%
Male	2245	86%	Medium	660	25%
**Education**			**Firm Age**		
No education	91	4%	1–5	532	21%
Primary School	209	8%	6–10	873	34%
Secondary school attempted and completed	1033	39.6%	11–15	457	18%
Vocational school	423	16%	16–20	306	12%
Some University	274	11%	21–25	162	6%
University Degree–undergrad	446	17%	26–30	140	5%
Masters	89	3%	31–35	64	2%
Ph.D. from Nigeria	24	1%	36–40	28	1%
Ph.D. from outside Nigeria	10	0.4%	>40	37	1%
**Entrepreneur Type**			**Industry**		
Opportunity	1726	66%	Manufacturing	1274	49%
Necessity	873	34%	Retail	565	22%
			Service	760	29%
**Respondent Age**			Financial **Constraints**		
30 years or less	440	17%	Access to financing as constraint	1936	75%
31–45	1176	45%	Access to financing not a constraint	663	25%
46–55	691	27%	Cost of finance as constraint	1967	76%
56 and more	292	11%	Cost of finance not a constraint	630	24%
**Years of managerial experience in industry**			Tax Constraint		
1–5 years	550	21%	Tax rate as constraint	1856	72%
6–10 years	919	35%	Tax rate not a constraint	741	28%
11–15 years	498	19%	Tax administration as constraint	1577	61%
16–20 years	325	13%	Tax administration not a constraint	1022	39%
21 and more	307	12%			
Position in firm			Institutional Constraint		
Founder/CEO	2125	82%	State courts constraint	1355	52%
Executive	474	18%	State court not constraint	1244	48%
			Federal courts constraints	1090	42%
			Federal courts not constraint	1509	58%
			Legal system corrupt	1387	53%
			Legal system not corrupt	1212	47%
**Owner Achievement Goal**			Bureaucratic Demands		
Growth Oriented	1952	75%	Laws and regulation are not consistent and predictable	1691	65%
			Laws and regulation are consistent and predictable	908	35%
Maintenance Oriented	647	25%	Spent more than 1% of executive time with state regulator per week	1721	66%
			Spent more than 1% of executive time with federal regulators per week	1200	46%
**Equity Ownership by Founder**					
Majority (>51%)	2508	97%			
Minority	91	3%			

### Distribution of evasive behavior in the sample

The distribution of evasive behavior by firm size and across industries is pervasive and presented in Table 2. Seventy-seven percent of firms under-reported revenue to evade income taxes, while 72% under-reported labor to evade wage taxes. Fifty-six percent reported paying bribes to government officials to win contracts, while 43% paid bribes to government officials with regard to customs duties, taxes, licenses, permits, regulation and services. Additionally, 39% paid both contract and service bribes while 63% under-reported both taxes.

**Table 2:** Illegal practices by Nigerian entrepreneurs (n = 2599).

	Industry							
Evasive and exploitative Practices	Manufacturing			Retail		Services		Total
	Small	Medium	Total	Small	Medium	Small	Medium	(n = 2599)
Under-report revenue tax only	814	268	1082	331	75	318	201	2007 (77%)
Under-report labour tax only	792	229	1021	268	75	314	183	1861 (72%)
Under-report both revenue and labour taxes	713	220	933	222	72	258	160	1645 (63%)
Pay contract bribe only	587	190	777	197	48	234	188	1444 (56%)
Pay service bribe only	468	136	604	135	41	212	132	1124 (43%)
Pay both contract & service bribes	450	131	581	119	40	143	130	1013 (39%)

### Empirical strategy

The research model was tested using Partial Least Squares [85]. PLS is particularly well suited for our analysis given its flexibility to handle constructs with both reflective and formative indicators [86, 87]. Because our model includes formative factors, rather than reflective, PLS was the most appropriate methodological approach [88]. PLS is robust with different scale types, such as metric, quasi-metric (e.g. Likert scales), dichotomous (e.g. dummy variables), and the assumption of equidistant scales, which are an assumption for certain analysis techniques, is not an assumption in PLS. Since we were not concerned with fulfilling the requirement of equidistance for the scales, the response categories were used as established in the WBES data.

Further, no distributional assumptions apply − the data may be non-normal, skewed, kurtotic, and the observations may be interrelated. As a result, it is robust to violations of multivariate normal distributions. Lastly, PLS allows more flexibility in analyzing theoretical models. Our PLS structural model will be evaluated by *R*^2^ of endogenous latent variables [86], effect size *f*^*2*^ [89], and by using the Stone-Geiser Q-square test for predictive relevance (Stone, 1974; Geiser, 1975).

Formative indicators have advantages from a theoretical and nomological perspective which make them suitable for theory-building: (1) formative measurement provides a means of modelling all the complex phenomena in our model from a diverse and potentially disparate set of observable indicators [87, 90], and (2) from a nomological perspective, formative measurement facilitates aggregation of these disparate indicators to the level of a holistic, single construct which improves parsimony and enhances the predictive value of our model [91]. We applied the criteria proposed by Diamantopoulos and Winklhofer [90] for index construction.

The measurement and structural models were estimated using 2000 bootstrap samples. Estimates reported are the mean of subsamples. Because our factors were formative, we used the regular PLS algorithm (rather than PLS Consistent). For the multigroup analysis, we used the built-in MGA functions in SmartPLS 3.2.8, including the metric invariance tests for invariance across groups for the measurement model. Table 3 summarizes the model constructs and scale of corresponding indicators.

**Table 3:** Construct definitions table.

Construct *Definition*	Measure	Scale
Evasive Culture *Perception of the prevalence of illegal activity in normal business*	It is common for establishments in this line of business to have to pay informal payments/gifts to get things done with regard to customs, taxes, licenses, regulations, etc.	Measured on a scale of 1 to 4 where higher number imply illegality is more pervasive in the industry
	Establishments in this line of business know in advance about how much informal payments/gifts is to get things done.	Measured on a scale of 1 to 5 where higher number reflects greater awareness of illegality in the industry.
Enactment of Evasive Behavior^a^ *Amount of bribes paid (exploitation) and taxes evaded by entrepreneurs*	When establishments like this one do business with the government, what percentage of the contract value would typically be paid in informal payments/gifts to secure the contract?	Amount of bribe to win government contract as % contract value (sales as proxy)
	We’ve heard that establishments are sometimes required to make gifts or informal payments to public officials to “get things done” with regard to customs, taxes, licenses, regulations, services etc. On average, what percentage of total annual sales do establishments like this one pay in informal payments/gifts to public officials for this purpose?	Amount of bribe to secure government services as % of sales (sales proxy)
	What percentage of total annual sales would you estimate a typical establishment in your sector of activity reports for tax purposes?	[100%—x%] (sales as proxy) is % of unreported revenues for tax purposes. Where x% is the answer provided by respondent
	What percentage of the total workforce would you estimate the typical establishment in your line of business declares for tax purposes?	[100%—x%] (wages as proxy) is % of unreported labor wages for tax purposes. Where x% is the answer provided by respondent.
Entrepreneurial Enablement *Factors contributing to the opportunity for entrepreneurs to exercise their agency to engage in evasive behaviour*.	What percentage of firm equity value owned by respondent?	% firm equity owned by respondent
	What is your goal for the next 5 years?	1 if goal is high growth, otherwise 0
	What is the highest level of education?	Years of education (1–10)
	What’s your age?	Age bracket of respondent. Measured on a scale of 1–4 where higher number implies higher age
	What’s your position in firm?	Owner (1), not (0)
	How many years in position?	Years in number
Economic Constraints *Level of financial and tax obstacles faced by entrepreneurs and their firms*	Do you think access to finance (e.g., collateral) present any obstacle to current operations of your business?	Scale: 1 = no obstacle to 5 = major obstacle
	Do you think cost of finance present any obstacle to current operations of your business?	
	Do you think tax rates present any obstacle to current operations of your business?	
	Do you think tax admin present any obstacle to current operations of your business?	
Legal Constraints *Degree to which the legal system and law enforcement are obstacles to doing business*	Do you think the functioning of the federal/state/local courts present any obstacle to current operations of your business?	Scale: 1 = no obstacle to 5 = major obstacle
	Do you think the crime, theft and disorder present any obstacle to current operations of your business?	
	Quality of legal system as obstacle to business?	
Bureaucratic Demands *Level of regulatory demands placed on entrepreneur activity*	What percentage of time spent dealing with state regulators?	Amount of time
	What percentage of time spent dealing with federal regulators?	Amount of time
	How uncertain is govt. regulation?	1 if regulation is uncertain and unpredictable, otherwise 0
Firm Performance *Amount of sales and employment growth over three years*	What was your sales in year 1, 2, and 3? What was your number of employees in year 1, 2, and 3?	Average change in sales revenue over three years (100xUS dollars) Average three-year change in full-time employees
Entrepreneur Type *Opportunity-driven entrepreneurs start new firms based on the discovery of unexploited or underexploited business opportunities while necessity-driven entrepreneurs do so because they lack other income options*.	a. What is the **main** reason for starting firm? Per our definition of we coded response ‘g’ as “Opportunity” and the others as “Necessity”	a. I could not find a job in the labor market b. The earnings in my previous job were too low c. I did not like my previous job (colleagues, tasks) d. I wanted to work flexible hours e. I wanted to work near home f. I wanted to improve/maintain the family income g. I wanted to exploit attractive market opportunities h. Personal satisfaction
Firm Size (Control) *Number of full-time employees*	Small (5–19 employees) Medium (20–100 employees)	Small = 0, medium = 1
Firm Age (Control) *Years since founding*	Number of years since founding	Number since founding

^a^ Evasive Culture and Evasive Behavior are related but distinct, as culture measures a perception of the general prevalence of bribery within the industry, while behavior refers to enacted bribery or tax evasion.

### Assessment of the measurement model

PLS estimates weights that measure the contribution of each formative indicator to the variance of the latent variable and are indicative of construct validity [92]. As shown in Table 4, all formative indicator weights are significant and explain a significant portion of the variance in the construct [93]. The negative weights shown for position/level of responsibility and equity ownership are significant and interpreted as follows: when tenure, age, educational level and achievement goal are otherwise equal, an increase in the level of responsibility and equity ownership is likely to reduce the opportunity for entrepreneurial agency to be exercised in the enactment of evasive behavior.

**Table 4:** Measurement weights of formative constructs.

Measures	Weights	t-statistic
Evasive Culture		
Pervasiveness of Evasive Behaviour	0.2697	4.630***
Entrepreneur’s Perception of Ease of Evasion	0.9067	31.074***
Enactment of Evasive Behavior		
Unreported Revenue	0.6296	8.2951***
Unreported Labour	0.4095	5.5228***
Log Contract Bribes Paid	0.2660	4.4939***
Log Service Bribes Paid	0.1327	2.6424**
Entrepreneurial Enablement		
Tenure	0.3354	4.4882***
Age	0.2928	4.0199***
Educational Level	0.3261	5.5139***
Position/level of Responsibility in the Firm	-0.3596	5.6114***
Equity Ownership	-0.5304	7.5477***
Achievement Goal	0.2709	5.9123***
Economic Constraints		
Financial Constraints (Access and Cost of Financing)	0.7052	9.6108***
Tax Constraints (Tax Rate and Tax Administration)	0.5564	6.6366***
Legal Constraints		
Federal Court	0.2655	4.0030***
State Court	0.6123	10.6894***
Legal System	0.3473	5.3009***
Environmental Hostility–Law Enforcement	0.5062	8.1682***
Bureaucratic Demands		
Time with State Leaders	0.4027	5.0116***
Time with Federal Leaders	0.6888	9.7409***
Regulation Uncertainty	0.1414	2.0910*
Firm Performance		
Employee Growth	0.6647	6.1171***
Sales Growth	0.6199	6.2987***

*p<0.05,

**p<0.01,

***p<0.001,

(ns) = not significant

Model fit was not assessed for the measurement model (or structural model), as model fit is a set of measures associated with and founded on the assumptions of covariance-based SEM. PLS, however, is not founded on these same assumptions, and therefore does not appropriately lend itself to measures of model fit [94].

### Mitigating endogeneity

Endogeneity can have various roots, such as measurement errors, simultaneous causality, common method variance, omitted variables, and unobserved heterogeneity [95–98]. Endogeneity is often evidenced in an estimate that is inflated due to a third unmeasured variable. For example, we may find that enactment of evasive behavior has a strong, positive relationship with firm performance. However, these two variables might both be affected by some third variable, such as firm’s financial resources to pay bribes to circumvent and entrepreneur’s social capital. Therefore, the size of the estimated relationship is likely inflated and not entirely due to the shared trait variance of these two variables.

In the context of our study, endogeneity with regards to main effect—Evasive Behavior → Firm Performance—may be the result of common performance antecedents, such as firm size [99] or firm age [100]. According to Ebbes, Papies [101] and Hult, Hair Jr [102], a fairly straightforward approach to reducing endogeneity is to simply control for such known antecedents that might explain some of that variance in the dependent variable [see also 97, 103]. This is exactly what we have done in our model with firm size and firm age–both of which demonstrate significant effects. We could not conduct comprehensive endogeneity test (e.g., the instrumental variable or Gaussian copula) due to data limitation. We acknowledge that the control variables may not necessarily account for all of endogeneity in the model [95, 104]. We reported this as a limitation when interpreting the results. We added control variables, which from a theoretical perspective, are likely to influence firm performance.

Endogeneity can also manifest as common method bias. We assessed common method bias using the modified [105] Lindell and Whitney [106] marker approach and the Kock [107] full collinearity approach. Both tests confirmed no significant bias due to a common source (all structural VIFs < 3.3). A bootstrap resampling (2000 samples) procedure was conducted to test for significance of hypothesized relationships. To further mitigate endogeneity and specific bias leading to inflated shared variance, we adopted a measurement strategy of including formative constructs in the model after applying Jarvis, MacKenzie [108]’s four primary guidelines in specifying formative constructs.

## Results

**Fig 2:**
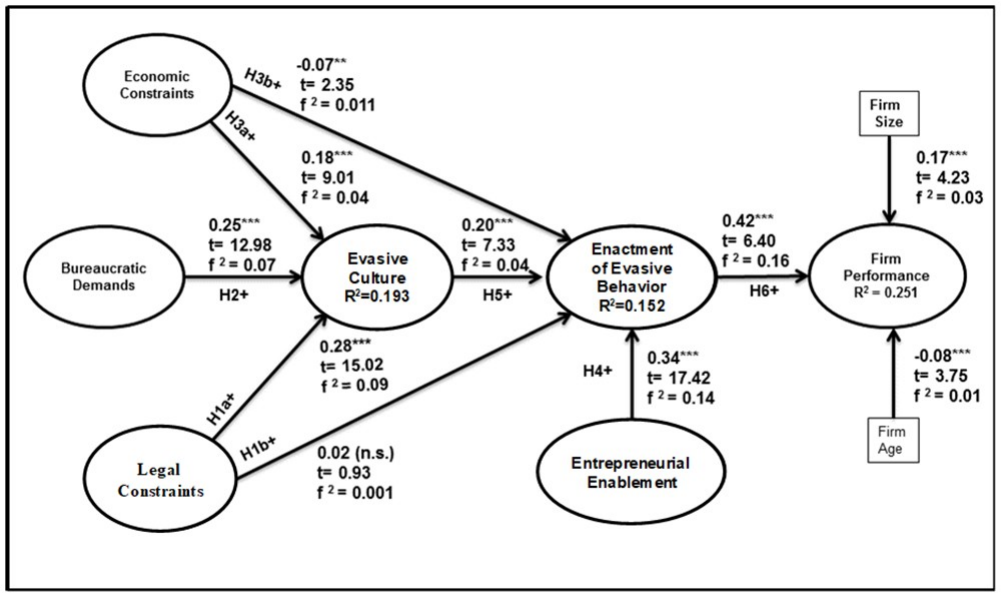
Structural analysis results.

Fig 2 presents our tested structural model, the estimated path coefficients, effect size (*f*^*2*^) and R^2^. We examined Cook’s D and found no influential cases, and checked VIFs (variance inflation factor) and found no substantial multicollinearity (all VIFs < 3.3).

The results support our main effect (H6): the enactment of evasive behavior is positively (and strongly) related to firm performance (β = 0.42, p < 0.0001). Entrepreneurial enablement (β = 0.34, p < 0.0001) and evasive culture (β = 0.20, p < 0.0001) are positively related to enactment of evasive behavior, confirming support for H4 and H5. In support of H1a, H2 and H3a, economic constraints (β = 0.18, p < 0.0001), bureaucratic demands (β = 0.25, p < 0.0001) and legal constraints (β = 0.28, p < 0.0001) have positive relationships with evasive culture. However, the relationship between legal constraints and enactment of evasive behavior is not significant (β = 0.02, p = 0.35); thus, H1b is not supported. Economic constraints (β = -0.07, p < 0.02) is negatively related to enactment of evasive behavior, though deemed not practically significant (*f*^*2*^ = 0.01). Thus, H3b is not supported. Multi-group analysis was conducted to determine whether the model was the same across entrepreneur type and industry sector. The result shows that entrepreneur type (‘necessity’ vs. ‘opportunity’) was not a useful moderator in explaining the relationship in the model. Thus, H7 is not supported. Therefore, our structural model is invariant between opportunity and necessity-driven entrepreneurs except for the effect economic constraint has on evasive culture.

We found that the model accounted adequately for variance in evasive culture (R^2^ = 0.193), enactment of evasive behavior (R^2^ = 0.152) and firm performance (R^2^ = 0.251). Moreover, legal constraints, bureaucratic demands and economic constraints demonstrate nomological validity through their strong relationship to evasive culture. Similarly, entrepreneurial enablement demonstrates nomological validity through its strong relationship with enactment of evasive behavior.

Our control variables capture effects due to firm size and age. Firm size, measured by number of employees, and defined as small (firms with less than 20 employees) and medium (firms with 20–100 employees) has a positive effect on firm performance, and firm age (measured as number of years since company founded) is negatively related to performance, consistent with extant literature [100].

### Predictive relevance and validity

The *f*^*2*^ statistic is based on the differences in R^2^ between two models − *with* and *without* the particular predictor. Cohen [89] recommends that effect sizes of 0.02, 0.15, and 0.35 be viewed as whether the predictor has a small, medium, or large effect. Fig 2 shows the effect size of our focal relationship from enactment of evasive behavior to firm performance is medium to large. Economic constraints, bureaucratic demands and legal constraints have small to medium effect sizes on evasive culture, and entrepreneurial enablement and evasive culture have small to medium effect sizes on enactment of evasive behavior. Lastly, a Stone-Geisser [109, 110] test statistic Q^2^, of 0.023 (Q^2^>0) indicated our model has predictive relevance.

## Discussion

Our paper builds on previous work and makes several important contributions. First, we show that evasive entrepreneurship has a pecuniary value to the entrepreneur who earns profits by exploiting existing institutional constraints through the mechanisms of bribery and tax evasion. Second, in contrast to Elert and Henrekson [3], who theorize evasive entrepreneurship from a formal institutional perspective, we combine both formal and informal institutions. We build on this literature by arguing differently and taking a broader view that both formal and informal institutions can combine to constrain and enable evasive behavior for profits. Given the social embeddedness of entrepreneurship [19, 111], the inclusion of informality (cultural factors) in our analysis of evasive entrepreneurship is an important and corrective adjustment to our understanding of entrepreneurial action and evasive entrepreneurship theorizing. Third, we expand the field of evasive entrepreneurship to include formal entrepreneurs. Prior to our study, informal entrepreneurs have been the dominant focus of evasive entrepreneurship theorizing. Fourth, we show that formal institutions (e.g., regulation) as well as informal institutions (e.g., evasive culture) are both important conditions in constraining and enabling enactment of evasive behavior. The literature on evasive entrepreneurship omits the important role of informal institutions. Finally, we show that in a developing economy like Nigeria, entrepreneurs don’t just evade, they exploit institutions for profit motive. In light of our contributions, we advance the entrepreneurship literature, entrepreneurial practice, institutional reforms, and development policymaking in emerging economies, such as Nigeria.

Our study provides empirical evidence–for the first time to our knowledge–that, in developing economies like Nigeria, evasive behavior by entrepreneurs pays off for the individual firm in the form of enhanced firm performance. We showed a direct positive relationship between the performance of firms whose owners pay bribes and evade taxes and the prevalence of those behaviors. Scholars have suggested that the legal system in economies like Nigeria is flawed and law enforcement is weak [61, 80], minimizing the probability of detection, prosecution and punishment. From the perspective of an evasive entrepreneur, the costs of illegal evasion (litigation costs, penalties, incarceration and associated social costs) are relatively low compared to the benefits of engaging in those acts. As has been observed about illegal evasive acts in general [112], positive reinforcement (or lack of negative enforcement) of illegal evasive behavior incentivizes more of it. Thus, if entrepreneurs perceive their desire for wealth and social success more efficiently achieved by means of evasive practices, evasive strategy will − and in Nigeria perhaps has − become a ‘best practice.’

Previous research on evasive behavior has focused narrowly on institutional constraints and government officers who demand bribes from entrepreneurs to circumvent them [e.g., 9, 32, 37, 62]. In contrast, we aimed a novel lens at business owners, revealing that (1) evasive entrepreneurs use bribery and tax evasion mechanisms to circumvent institutional constraints to improve firm performance; (2) economic constraints, bureaucratic demands and legal obstacles predict the evasive culture and; (3) the economic value of evasion derives from the extent entrepreneurs exercise agency to discover, evaluate and exploit it for private gain.

Baumol theorized that entrepreneurial individuals channel their effort in different directions depending on the quality of prevailing formal institutions. Where institutional rules of the game incentivize innovation, entrepreneurs engage in innovative entrepreneurship; where the institutional framework incentivizes rent-seeking behavior such as bribery and tax evasion, entrepreneurs will enact evasive entrepreneurship [1, 113–115].

In proposing these implications, we also caution that our findings may not be generalizable across all emerging economies. Our analysis focused solely on the Nigerian sample, and did not attempt any weighting scheme to produce estimates at the population level. Additionally, the World Bank data we used was self-reported using a cross-sectional design and thus the direction of causality cannot be fully substantiated, though we employed three-year averages for the firm performance measure. In our case, only entrepreneurs themselves have knowledge of their own illegal evasive behavior. Additionally, drawing on existing research, we identified a set of key variables from the WBES, but we recognize there may be other factors and measures that impact enactment of evasive behavior and firm performance and these should be considered in future research–we simply used those variables available to us in the WBES dataset.

We opted to use data from 2009, as this was the most complete available at the time. The survey instrument changed in subsequent iterations, and these changes removed or altered certain measures (such as culture and necessity vs. opportunity entrepreneurship), preventing us from testing the desired theory with newer iterations of this data. Therefore, we chose to use an older dataset to measure more precisely. However, this creates a limitation, particularly as the Nigerian economy has moved towards more global inclusivity since the global financial collapse of 2008 and then was rebased in 2014. While these events and actions may have bearing on entrepreneurial behavior, we expect these institutional issues to be robust to these events in the long run. Nevertheless, without a comparative analysis, we cannot be certain our theory is generalizable to systems dissimilar to the context we analyzed with the data we had available.

Due to data limitations, we could not conduct comprehensive endogeneity tests (e.g., the instrumental variable or Gaussian copula). As such, the approach we adopted may not necessarily account for all of endogeneity in the model. This is a limitation when interpreting the results. As noted by Tonoyan et al [12]: “The problem of endogeneity could not be solved in this study because of the missing “instrument variables” in the WBES. Future work should ideally determine the direction of causality, while drawing on different sources of data and utilizing appropriate instrumental variables” (p. 825).

However, regarding reverse-causality, the view that high performing firms pay bribes because they are more likely to be extorted by bureaucrats due to their financial attractiveness is inconclusive. There is even strong evidence that underperforming firms are more pressured to engage in bribery and tax evasion to improve firm performance than high performing firms [7]. While Clarke and Xu [116] and Fredriksson and Svensson [117] find a positive relationship, more recent studies by Şeker and Yang [118] and Wu [119] did not find such a relationship. Rather, firms experiencing slower growth were just as likely to engage in bribery as the fast-growing firms. Additionally, Ufere, Perelli [7] found that the bargaining power of underperforming firms is smaller than for high performing firms, making them easier targets for bureaucrats to extort.

Additionally, our measure for firm performance is limited by the available data. We used growth in sales and employees. While this is not ideal, these were the two variables that best represented firm performance within that dataset. Our measure of firm performance is similar to many influential papers which measure the effect of corruption on firm performance [c.f.., 116, 120–124].

Lastly, we did not examine informal entrepreneurship in our study due to the scope of data collected by World Bank. Data on informal entrepreneurship simply was not accounted for in this dataset. Although there is a healthy body of literature already on informal entrepreneurship, future studies might benefit from comparing and contrasting evasive behavior by formal and informal entrepreneurs in institutionally constrained environments.

In summary, our findings reveal that firm performance is positively influenced by evasive behavior enacted by entrepreneurs who proactively discover and deliberately exploit institutions for private gain. Rewards associated with evasive entrepreneurship may institutionalize this type of entrepreneurship as normative. These results, and our finding that evasive culture increase with bureaucratic and regulatory intervention in entrepreneurial activities, have implications for policymakers who seek to deter bribery and tax evasion through expanding government oversight. Excessive intervention may, paradoxically, but powerfully, provoke evasive behavior. Previous research has often cast entrepreneurs as victims of institutional constraints and bribe demanding and high-taxing officials, but our results highlight their self-interested engagement in perpetuating illegal evasion to boost firm performance.

## Conclusion

While previous research has focused on cultural, political, institutional and economic factors that affect entrepreneurial behavior, the role of entrepreneurial agency (enabled through personal factors) has been largely ignored. Empirical evidence of evasive proclivity by entrepreneurs and the positive effects on the performance of their firms should provoke the concern of policy makers, government officials and institutions that have historically focused on institutional governance to stem bribery and tax evasion. Exposing entrepreneurs as agents of illegal evasive behavior offers a non-traditional perspective of the problem and invites alternative thinking about it. The indisputably critical role of entrepreneurs in transforming emerging economies, such as those in Africa, provides an extraordinary opportunity for researchers to produce scholarly work with practical relevance and high societal impact.
